# Hesperetin Promotes Cisplatin−Induced Apoptosis of Gastric Cancer *In Vitro* and *In Vivo* by Upregulating *PTEN* Expression

**DOI:** 10.3389/fphar.2020.01326

**Published:** 2020-08-27

**Authors:** Pengzhan He, Jingjing Ma, Yinghui Liu, Huan Deng, Weiguo Dong

**Affiliations:** ^1^Department of Gastroenterology, Renmin Hospital of Wuhan University, Wuhan, China; ^2^Central Laboratory, Renmin Hospital of Wuhan University, Wuhan, China; ^3^Key Laboratory of Hubei Province for Digestive System Disease, Wuhan, China; ^4^Department of Geriatrics, Renmin Hospital of Wuhan University, Wuhan, China

**Keywords:** gastric cancer, hesperetin, cisplatin, apoptosis, phosphatase and tensin homolog, mitochondrial pathway

## Abstract

As one of the most common malignant gastrointestinal tumors, gastric cancer (GC) has a high incidence and poor prognosis. Cisplatin (DDP) is often used as chemotherapy for advanced GC; however, the high incidence of drug resistance remains a problem. The use of several anti-tumor drugs as combined chemotherapy is an effective strategy. Hesperetin has anti-tumor ability *via* its pro-apoptotic effect on various human cancers, both *in vitro* and *in vivo*, with no significant toxicity. However, a combination of DDP and hesperetin in GC has not been reported. The present study aimed to investigate the *in vitro* and *in vivo* chemosensitization effect and mechanism of hesperetin-augmented DDP-induced apoptosis of GC. The proliferation of GC ty -60cells was inhibited significantly in a time and dose-dependent manner by combined treatment of DDP with hesperetin. Hesperetin markedly increased DDP-induced apoptosis of GC cell lines. In a xenograft tumor mouse model, markedly better tumor suppression was observed after treatment with DDP plus hesperetin compared with that of either agent alone. Additionally, the combination of DDP and hesperetin remarkably increased the expression levels of phosphatase and tensin homolog (PTEN) and Cytochrome C (Cyt C), and significantly decreased the levels of phosphorylated protein kinase B (p-AKT) and CyclinD1. DDP and hesperetin also induced significant increases in apoptosis inducing factor (AIF), BCL2 associated X, apoptosis regulator (BAX), cleaved caspase-9, and cleaved caspase-3, and decreased B-cell lymphoma 2 (BCL2), caspase-9, and caspase-3 levels. Thus, we demonstrated that hesperetin could inhibit the phosphatidylinositol-4,5-bisphosphate 3-kinase (PI3K)/AKT signaling pathway and induce the mitochondrial pathway *via* upregulating PTEN expression, thereby significantly enhancing DDP’s anti-tumor effect on GC. Hesperetin is a potential chemotherapeutic agent for GC and merits further clinical investigation.

## Introduction

Gastric cancer (GC) is a highly prevalent type of malignant tumor and is the third most common cause of cancer-related death worldwide. Based on the latest data from GLOBOCAN 2018, the number of new cases of GC worldwide is estimated to exceed 1,000,000 in 2018, ranking fifth among all cancers; the number of deaths is estimated as 783,000, second only to lung cancer and liver cancer (Bray et al., 2018). The areas with the highest incidence are East Asia, Eastern Europe, and South America. In China, the incidence and mortality of GC have decreased; however, it remains an important disease that seriously endangers the health of the Chinese population (Chen et al., 2016). Anatomically, GC is divided into two types: True gastric adenocarcinoma (non-cardiac GC) and gastro-esophageal-junction adenocarcinoma (cardiac GC). Currently, surgery, chemotherapy, radiotherapy and their combinations are the main treatments for GC. At diagnosis, about 70% of patients with GC are already in the advanced stage because the early symptoms are not typical, which markedly limits surgical and radiotherapy efficacy (Song et al., 2017). Over the past few decades, conventional chemotherapeutic drugs, such as cisplatin (DDP) and 5-fluorouracil, have brought great clinical benefits to patients with advanced GC. However, because of drug resistance and cytotoxicity, clinical efficacy has gradually deteriorated (Wagner et al., 2017). Hence, to overcome drug resistance and improve the curative effect of chemotherapeutic drugs, it is necessary to identify natural anticancer drugs with low toxicity and high efficiency, and to explore new combinations of chemotherapy regimens.

Hesperetin (3’,5,7-trihydroxy-4’-methoxyflavone), is a member of the dihydroflavonoids and is the glycosyl ligand of hesperidin, which mainly exists in fruits such as citrus. Currently, hesperetin has been proven to have a wide range of pharmacological effects, including anti-inflammatory, antioxidant, antiviral, and cardiovascular protection (Parhiz et al., 2015). Previous studies have also revealed that it can significantly inhibit proliferation and promote apoptosis in various types of cancer, including prostate cancer, non-small cell lung cancer, breast cancer, hepatocellular carcinoma, and cervical cancer (Alshatwi et al., 2013; Sak et al., 2018; Xia et al., 2018; Sheokand et al., 2019; Zhang et al., 2019). More importantly, there is no report indicating that hesperetin is significantly toxic to normal cells (Sak et al., 2018; Ferreira de Oliveira et al., 2019). Hesperetin exerts its anti-tumor activity through various mechanisms, especially by interfering with a variety of carcinogenic signaling pathways, promoting mitochondrial apoptosis pathways, and affecting the generation of intracellular reactive oxygen species (Roohbakhsh et al., 2015; Ferreira de Oliveira et al., 2019). In addition, an interesting study showed that hesperetin could also inhibit the phosphatidylinositol 3-kinase (PI3K)/protein kinase B (AKT) signaling pathway by upregulating the expression of phosphatase and tensin homolog (PTEN), thus inhibiting cell proliferation (Li et al., 2017). Moreover, PTEN is likely to play an important role in the occurrence and development of GC (Guo et al., 2013; Shen et al., 2018; Cai et al., 2019). To date, there has been no report on the anti-tumor effect of DDP combined with hesperetin on GC or whether PTEN is involved in its anti-cancer process.

Previously, our research group demonstrated that hesperetin could inhibit the proliferation and promote apoptosis of GC cells *in vitro* and *in vivo* (Zhang et al., 2015). The present study aimed to investigate the effect of hesperetin on apoptosis and growth inhibition induced by DDP in GC and its potential mechanism.

## Materials and Methods

### Cell Culture

The China Center for Type Culture Collection (CCTCC) provided the normal cell line (GES-1) and the human GC cell lines (HGC-27, SGC-7901, and MGC-803), which were cultured in RPMI 1640 medium (Gibco, Grand Island, NY, USA) and DMEM/F-12 medium (1:1) (HyClone, Logan, UT, USA) with 10% fetal bovine serum (FBS) (Gibco), with 1% solution of antibiotics (penicillin at 100 U/ml and streptomycin at 100 g/ml) (Beyotime, Jiangsu, China). The DDP-resistant HGC-27 cell line (HGC-27/DDP) was previously established by our team *via* gradually increasing the exposure concentration of DDP to HGC-27 [31695431]. The cells were incubated in 5% CO_2_ at 37°C in a humidified incubator.

### Antibodies and Reagents

Hesperetin (> 98% purity) and DDP were obtained from Sigma-Aldrich (St. Louis, MO, USA). A 200 mM stock solution of hesperetin was prepared by dissolving it in absolute dimethyl sulfoxide (DMSO), which was stored at -20°C. A 4 mg/ml stock solution of DDP was prepared by dissolving it in normal saline, which was stored at -20°C. Lipofectamine^®^ 2000 Transfection Reagent (Thermo Fisher Scientific, Waltham, MA, USA) was stored at 4°C. The Shanghai Genechem biotechnology company (Shanghai, China) provided plasmids PTEN-shRNA (PTEN-RNAi-38319) and PTEN-NC (negative control, CON077), which were maintained in glycerol bacterial cultures at -20°C.

Rabbit monoclonal antibodies including those recognizing PTEN, AKT, phosphorylated (p)-AKT, BCL2 associated X (BAX), B-cell lymphoma 2 (BCL2), apoptosis inducing factor (AIF), Cytochrome C (CytC), CyclinD1, matrix metalloproteinase (MMP)-2, MMP-9, cleaved caspase-3, cleaved caspase-9, caspase-9, caspase-3, and glyceraldehyde 3-phosphate dehydrogenase (GAPDH) were obtained from Cell Signaling Technology (Danvers, MA, USA). The antibodies were used at a working concentration of 1:1000 and were stored at 4°C. The secondary antibodies were purchased from LI-COR (Lincoln, NE, USA) and used at a dilution ratio of 1:10,000.

### Transfection

Plasmids PTEN-shRNA and PTEN-NC, which contain green fluorescent protein (GFP) and anti-puromycin genes, were extracted from glycerol bacteria and purified according to the manufacturer’s protocols. GC cells were cultured in DMEM/F-12 with 10% FBS in 6-well plates until they reached 70%–80% confluence. The cells were then starved for 2 h in DMEM/F-12 without FBS. According to the manufacturer’s protocols, 4 μg of PTEN-shRNA or PTEN-NC were transfected into cells using Lipofectamine 2000. Twenty-four hours later, 0.5 µg/ml puromycin in DMEM/F-12 with 10% FBS was added to the transfected cells to assess stable transfection. Ultimately, HGC-27/SGC-7901/MGC-803-PTEN and HGC-27/SGC-7901/MGC-803-NC were obtained successfully. The transfection efficiency was observed indirectly *via* observing GFP under a fluorescence microscope. Downregulation of the PTEN level was evaluated quantitatively using western blotting.

### *In Vitro* Cell Proliferation Assay

Cell proliferation and viability were assessed quantitatively using a Cell Counting Kit-8 (CCK-8, Beyotime, Shanghai, China) according to the manufacturers’ instructions. All GC cells were seeded into 96-wells plates (at 8 × 10^3^ cells/well) and cultured for 24 h. The SGC-7901, HGC-27, MGC-803, HGC-27/DDP and GES-1 cells were initially treated with different concentrations of hesperetin (0, 50, 100, 200, 400, 600, and 800 µM), different concentrations of DDP (0, 0.5, 1, 2, 4, 6, and 8 µg/ml), or a combination of DDP (0, 0.5, 1, 2, 4, 6, and 8 µg/ml) and hesperetin (50 µM or 0, 50, 100, 200, 400, 600, 800 µM). Subsequently, the cells were incubated at 37°C with 5% CO_2_ for another 24 h. The supernatant was removed, 10 µl of CCK-8 solution was added to each well, and the cells were cultured for a further 2 h. To determine the interaction between the two drugs, the combination index (CI) and fraction affected (Fa)-CI plots were obtained using CompuSyn software (ComboSyn, Inc, Paramus, NJ, USA) based on the Chou-Talalay method. CI < 1, CI = 1 and CI > 1 represent synergistic, additive, and antagonistic effects, respectively.

Transfected GC cells and NC group cells were treated with DDP (0, 0.5, 1, 2, 4, 6, and 8 μg/ml) or the combination of hesperetin (50 μM) with DDP (0, 0.5, 1, 2, 4, 6, and 8 μg/ml) for 24 h. Cell viability was then examined using the CCK-8 assay. Finally, the absorbance of the colored formazan reaction product was measured at 450 nm using a microplate reader (Victor3 1420 Multilabel Counter, Perkin Elmer, Waltham, MA, USA). Control cells were incubated in DMEM containing 10% CCK-8. GraphPad Prism software (GraphPad Software, Inc, La Jolla, CA, USA) was used to calculate the half maximal inhibitory concentration (IC50), and each experiment was repeated 3 times.

### Transwell Invasion Assay

Trypsin Solution (Biosharp, Anhui, China) was used to digest the GC cells and transfected GC cells and 100 µl of the cell suspension (approximately 2×10^4^ cells) was seeded into the upper chamber of a Transwell insert (Corning Costar Corp, Corning, NY, USA), which had 8 µm pores and was precoated with Matrigel (BD Biosciences, San Jose, CA, USA). Medium supplemented with 25% FBS (600 µl) filled the lower chamber and the apparatus was left overnight. The next day, the medium in the lower chamber was exchanged for medium with DDP (4 μg/ml), hesperetin (200 μM) or DDP (4 μg/ml) plus hesperetin (200 μM). After a further 24 h, the cells in the insert were fixed using 4% paraformaldehyde for 15 min and stained using 0.1% crystal violet. Eight random fields under the microscope were used to calculate the number of invaded cells.

### Wound-Healing Assay

Cells in fresh medium were placed into a 6-well plate chamber (1×10^5^ cells/well) and incubated for 24 h. When the cells were 80% confluent, a scratch in the cell monolayer was made using a 200-µl pipette tip. Phosphate-buffered saline (PBS) was used to remove floating debris and the wound was photographed immediately (0 h). The cells were then cultured in DMEM/F-12 medium with 3% FBS together with DDP (4 μg/ml), hesperetin (200 μM), or DDP (4 μg/ml) plus hesperetin (200 μM). The wounds were photographed at 24 and 48 h to measure the extent of wound healing.

### Detection of Apoptotic Cells Using the Hoechst 33258 Assay

Hoechst 33258 Staining Kit (Beyotime, Shanghai, China) was used to detect the morphological features of apoptotic cells. Exponentially growing GC cells were placed into sterile 6-well plates at 1×10^5^ cells/well and incubated for 24 h. Subsequently, the cells in the wells were treated with hesperetin (200 μM), DDP (4 μg/ml), or hesperetin + DDP (200 μM + 4 μg/ml) for another 24 h. Ultimately, the cells were stained with Hoechst 33258 according to the manufactures’ instructions. Additionally, transfected GC cells were also exposed to hesperetin + DDP (200 μM + 4 μg/ml) for 24 h and stained as described above. The morphological features of the apoptotic cells were observed under a fluorescent microscope (BX51, Olympus, Tokyo, Japan). The ratio of the apoptotic cell number to the total cell number defined the apoptosis ratio.

### Apoptosis Detection Using Annexin V-PE/7-AAD Double Staining

The percentage of apoptotic cells was determined using an Annexin V-PE/7-AAD kit (MultiSciences, Hangzhou, China) together with flow cytometry (FACSCalibur, Becton Dickinson, Franklin Lakes, NJ, USA). Cells seeded in 6-well plates were treated in culture with hesperetin (200 μM), DDP (4 µg/ml) or hesperetin + DDP (200 μM + 4 μg/ml) for 24 h. The adherent cells were collected, washed 2 times with cold PBS, and then co-stained with 10 µl 7-AAD and 5 µl Annexin V-PE in the dark for 15 min at room temperature before analysis using flow cytometry. Additionally, transfected GC cells were also exposed to hesperetin + DDP (200 μM + 4 μg/ml) for 24 h and stained as described above. Density plots were used to show four cell populations, e.g., live, early apoptotic, necrotic, and late apoptotic and dead, according to their various fluorescence characteristics: Live cells (PE and 7-AAD negative), early apoptotic cells (PE positive and 7-AAD negative), late apoptotic cells (PE and 7-AAD positive), and necrotic cells (PE negative and 7-AAD positive).

### Western Blotting Analysis

Western blotting was used to determine protein levels. Seeded in 6-well plates and grown for 24 h, GC cells were treated with hesperetin, DDP, or hesperetin + DDP as described in section *In Vitro Cell Proliferation Assay*. Total proteins were extracted from subcutaneous tumor tissues from nude mice or GC cells. Protein concentrations were determined using a BCA Protein Assay Kit (Beyotime), according to the manufactures’ instructions. SDS-PAGE was used to separate the cellular proteins, which were then electrotransferred onto polyvinylidene difluoride (PVDF) membranes (Millipore, Billerica, MA, USA). After blocking the membranes using 5% non-fat dry milk in TBST for 1.5 h, the blots were incubated at 4°C overnight with various primary rabbit antibodies. The membranes were washed with TBST 3 times (10 min each time), and then incubated with secondary antibodies at room temperature for 1 h. The membranes were then washed with TBST 3 times. Finally, a two-color Odyssey infrared imaging system (LI-COR Biosciences) was used to scan the membranes. The level of GAPDH was used to normalize the specific protein levels on the same PVDF membrane.

### *In Vivo* Xenograft Tumor Models

Serum-free DMEM was used to wash the collected HGC-27 cells, which were suspended in 100 μl of PBS, and then implanted into the dorsal area, subcutaneously, of male BALB/c nude mice (5 weeks old, Beijing Vital River Laboratory Animal Technology, China). After the tumors grew to 100–150 mm^3^, we randomly divided the nude mice into four groups (n = 6 per group), which were treated with intraperitoneal injection of normal saline, hesperetin (5 mg/kg), DDP (5 mg/kg), or hesperetin + DDP (both 5 mg/kg), respectively, every 2 days. The size of the tumor was determined using Vernier calipers by two researchers every 2~3 days. The tumor volume (TV) was calculated using the formula: TV (mm^3^) = 0.5 × d^2^ × D (where d and D are the shortest and longest diameters, respectively). In addition, 2–3 times per week, all the mice were weighed. After 30 days of treatment, the tumors were excised, weighed, and analyzed using the terminal deoxynulceotidyl transferase nick-end-labeling (TUNEL) assay. Renal and liver function were assessed by detecting of the levels of blood urea nitrogen (BUN), alanine aminotransferase (ALT), serum creatinine (Cr), and aspartate aminotransferase (AST).

All the animal research procedures were performed according to the institutional ethical standards and/or those of the national research committee and according to the 1964 Helsinki declaration and its later amendments or comparable ethical standards. The Ethics Committee of Renmin Hospital of Wuhan University approved the study protocol.

### HE Staining and TUNEL Assay

For hematoxylin and eosin (HE) staining, extracted tissues were fixed, dehydrated using an ethanol gradient, and embedded in paraffin. The tumor tissues from mice were cut into 4-µm sections and stained using HE. To detect apoptotic cells, a TUNEL assay was performed using an apoptosis detection kit (Roche Applied Science, Basel, Switzerland). An optical fluorescence microscope (Olympus) was used to observe the specimens. Positive cells were identified, counted, and analyzed.

### Statistical Analysis

All data were analyzed using SPSS 20 for Microsoft Windows (IBM Corp., Armonk, NY, USA) and presented as the mean ± SD. Differences among groups were determined using analysis of variance (ANOVA). Statistical significance was accepted at a value of *P* < 0.05.

## Results

### Hesperetin Enhances the Growth Inhibition of GC Cells Induced by DDP, Which Could Be Reversed by *PTEN* Downregulation

**Figure 1:**
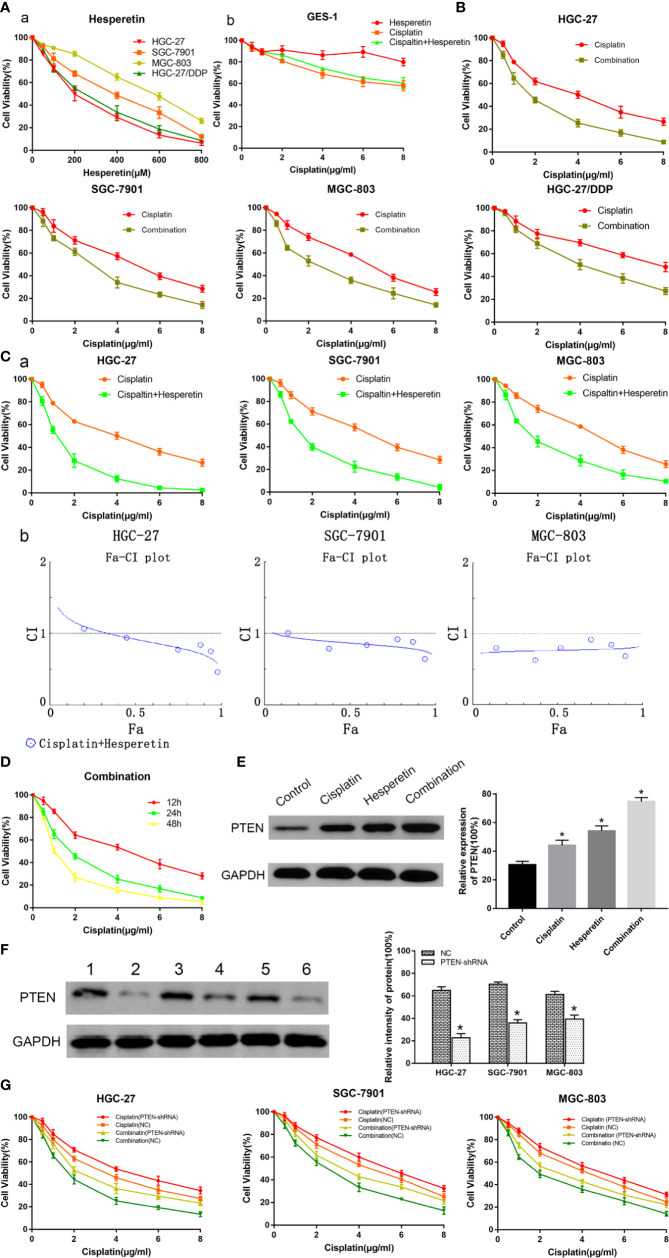
Evaluation of the inhibitory effect of hesperetin, or cisplatin, or both, on the growth of GC cells using a CCK-8 kit. **(A)** a. GC cells (HGC-27, SGC-7901, MGC-803, and HGC-27/DDP) were treated with hesperetin (0, 50, 100, 200, 400, 600, and 800 µM). b. GES-1 cells were treated with cisplatin (0, 0.5, 1, 2, 4, 6, and 8 µg/ml), the different concentrations of hesperetin as described above and a combination of cisplatin and hesperetin for 24 h. **(B)** GC cells were treated with cisplatin and a combination of cisplatin and hesperetin (50 µM) for 24 h, respectively. **(C)** a. GC cells were treated with cisplatin and a combination of cisplatin and hesperetin (0, 50, 100, 200, 400, 600, and 800 µM) as described above for 24 h, respectively. b. Compusyn software was used to define the type of drug-combination effect. **(D)** HGC-27 cells were treated with the same concentration of cisplatin combined with hesperetin for 12, 24, or 48 h. **(E)** HGC-27 cells were incubated with the control, 4 μg/ml cisplatin, 200 μM hesperetin, or 4 µg/ml cisplatin + 200 μM hesperetin, and the levels of PTEN in GC cells were measured using western blotting. **(F)** The transfection efficiency of *PTEN* in GC cells was identified using western blotting. Lane 1 HGC-27/NC; Lane 2 HGC-27/PTEN-shRNA; Lane 3 SGC-7901/NC; Lane 4 SGC-7901/PTEN-shRNA; Lane 5 MGC-803/NC; Lane 6 MGC-803/PTEN-shRNA. *P < 0.05 *vs.* the corresponding negative control (NC) groups. **(G)** Transfected GC cells were treated with cisplatin (0, 0.5, 1, 2, 4, 6, and 8 µg/ml), and a combination of cisplatin and hesperetin (50 µM) as described above for 24 h, respectively. All the above data are shown as the mean ± SD from an average of three experiments.

GC cells and GES-1 cells were treated using different concentrations of hesperetin, DDP, or their combination for 24 h. When the concentration of hesperetin was 50 µM, it had no significant effect on the viability of about 90% GC cells (**Figure 1Aa****)**. And below 600 µM, hesperetin has no obvious toxicity to GES-1 (**Figure 1Ab****)**. The combination of hesperetin and DDP did not enhance the toxicity of DDP to normal cells, but decreased the toxicity of DDP by reducing the IC50 of DDP. In addition, the results showed that hesperetin enhanced the DDP-induced growth inhibition of GC cells and the combination of hesperetin and DDP further enhanced growth inhibition of GC cells in a concentration-dependent manner **(****Figures 1B, Ca****)**. We used CompuSyn software to generate Fa-CI plots, showing that DDP and hesperetin synergistically inhibited the proliferation and viability of GC cells. In GC cells co-treated with DDP and hesperetin (50 µM), the IC50 value of DDP was significantly lower than that of DDP alone **(****Table 1****)**. Among the cell lines, HGC-27 was the most sensitive to hesperetin. The IC50 values of DDP and hesperetin in HGC-27 at 24 h were about 4 µg/ml and 200 µM, respectively. We chose HGC-27 and SGC-7901 cells for subsequent experiments. We next exposed HGC-27 cells to the same concentration of DDP combined with hesperetin for 12, 24, and 48 h, which showed a time-dependent inhibition of GC cell proliferation. After treatment with DDP, hesperetin, and DDP combined with hesperetin, we observed a significant increase in the expression of PTEN **(****Figure 1E****)**.

**Table 1:** Summary of IC50 values of cisplatin in GC and transfected GC cells^1^.

Cell type	IC50 ± SD (μg/ml)^2^	
	Cisplatin	Hesperetin + Cispaltin
HGC-27	3.85 ± 0.08	1.71 ± 0.13*
HGC-27/NC	3.66 ± 0.15	1.65 ± 0.09^#^
HGC-27/PTEN-shRNA	4.02 ± 0.11^#^	2.09 ± 0.08^&^
SGC-7901	4.36 ± 0.22	2.46 ± 0.29*
SGC-7901/NC	4.48 ± 0.32	2.38 ± 0.14^#^
SGC-7901/PTEN-shRNA	5.03 ± 0.06^#^	2.67 ± 0.23^&^
MGC-803	4.59 ± 0.16	2.07 ± 0.05^#^
MGC-803/NC	4.51 ± 0.07	1.83 ± 0.13*
MGC-803/PTEN-shRNA	4.82 ± 0.11^#^	2.34 ± 0.17^&^

^1^GC and transfected GC cells were exposed to cisplatin (0, 0.5, 1, 2, 4, 6, 8 μg/ml) and a combination of hesperetin (50 μM) and cisplatin (0, 0.5, 1, 2, 4, 6, 8 μg/ml) for 24 h, respectively.

^2^All the above data are presented as the mean ± standard deviation (SD) from the average of three experiments.

*P < 0.05 versus corresponding GC cells exposed to cisplatin alone.

^#^P < 0.05 versus corresponding GC/NC cells incubated with cisplatin alone.

^&^P < 0.05 versus corresponding GC/NC cells incubated with a combination of hesperetin and cisplatin.

GC cells were stably transfected with PTEN-shRNA to downregulate *PTEN* expression, or were transfected with NC plasmid as a control. Western blotting was used to assess the transfection efficiency **(****Figure 1F****)**. Transfected GC cells were treated with DDP (0, 0.5, 1, 2, 4, 6, and 8 μg/ml), and a combination of DDP (0, 0.5, 1, 2, 4, 6, and 8 μg/ml) + hesperetin (50 μM), respectively. Hesperetin could enhance the growth inhibition induced by DDP; however PTEN downregulation reversed the effects on GC cells of hesperetin combined with DDP **(****Figure 1G****)**. Moreover, the results showed that hesperetin reduced the IC50 of DDP, while downregulating PTEN expression reversed this effect **(****Table 1****)**.

### Hesperetin Promotes the Inhibitory Effect of DDP on the Invasion and Migration of GC Cells

**Figure 2:**
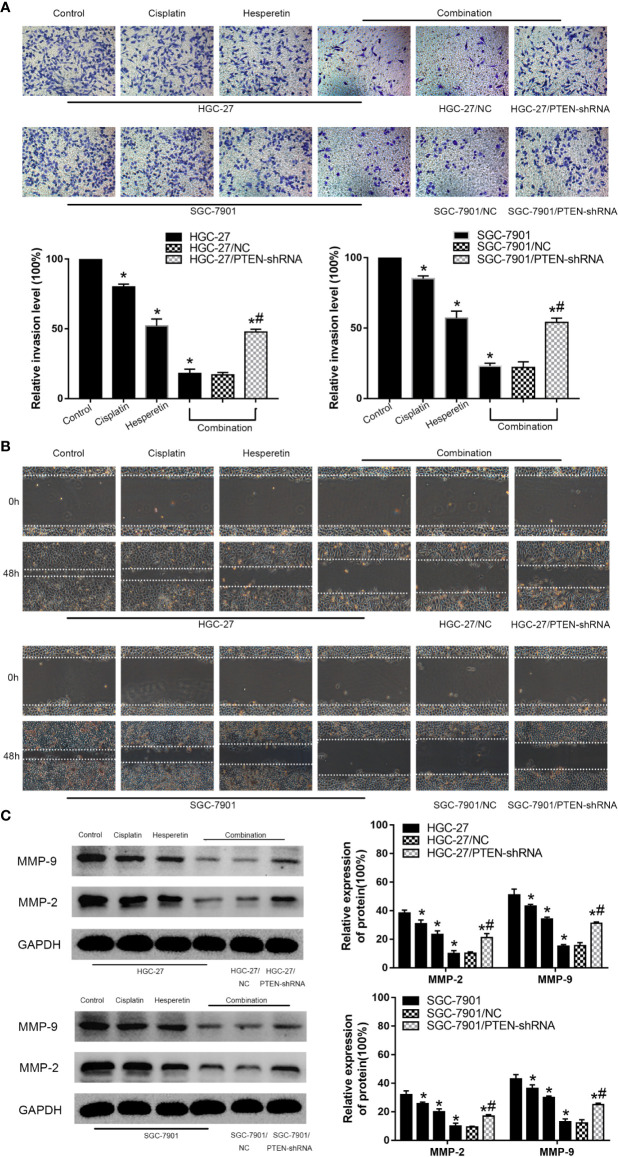
Effects of hesperetin and DDP on the invasion and migration of GC cells using the Transwell invasion assay and the wound-healing assay. **(A)** Original magnification: 200×. HGC-27 (SGC-7901) cells were treated with control, 4 μg/ml cisplatin, 200 µM hesperetin, or 4 μg/ml cisplatin + 200 µM hesperetin; HGC-27(SGC-7901)/NC and HGC-27(SGC-7901)/PTEN-shRNA were treated with 4 μg/ml cisplatin + 200 µM hesperetin. Quantitative analysis of the average invasive cell numbers in each group. *P < 0.05 *vs.* the control group, *^#^*P* < 0.05 *vs.* the NC group. **(B)** Cells were incubated with control, cisplatin, hesperetin, or cisplatin + hesperetin described in **(A)** above. **(C)** The levels of MMP-9 and MMP-2 were measured using western blotting and quantitative analysis of the proteins was performed. *P < 0.05 *vs.* the control group, *^#^*P* < 0.05 *vs.* the NC group. All the above data are the mean ± SD from an average of three experiments.

To explore the effects of hesperetin and DDP on the invasion and migration of HGC-27 and SGC-7901, Transwell invasion assays and wound-healing assays were conducted. Meanwhile, the levels of MMP-2 and MMP-9 were detected using western blotting. The results showed that DDP combined with hesperetin significantly inhibited GC cells invasion and migration compared with the other groups. The downregulation of PTEN attenuated the inhibitory effect of the combination treatment on cell invasion and migration **(****Figures 2A–C****)**. Furthermore, the levels of the tumor invasion-related proteins MMP-2 and MMP-9 decreased significantly after treatment with hesperetin and DDP, which was reversed by downregulation of PTEN (^#^*P* < 0.05) **(****Figure 2C****)**.

### Hesperetin Sensitizes GC Cells to the Apoptosis Induced by DDP, Which Is Related to PTEN Expression

**Figure 3:**
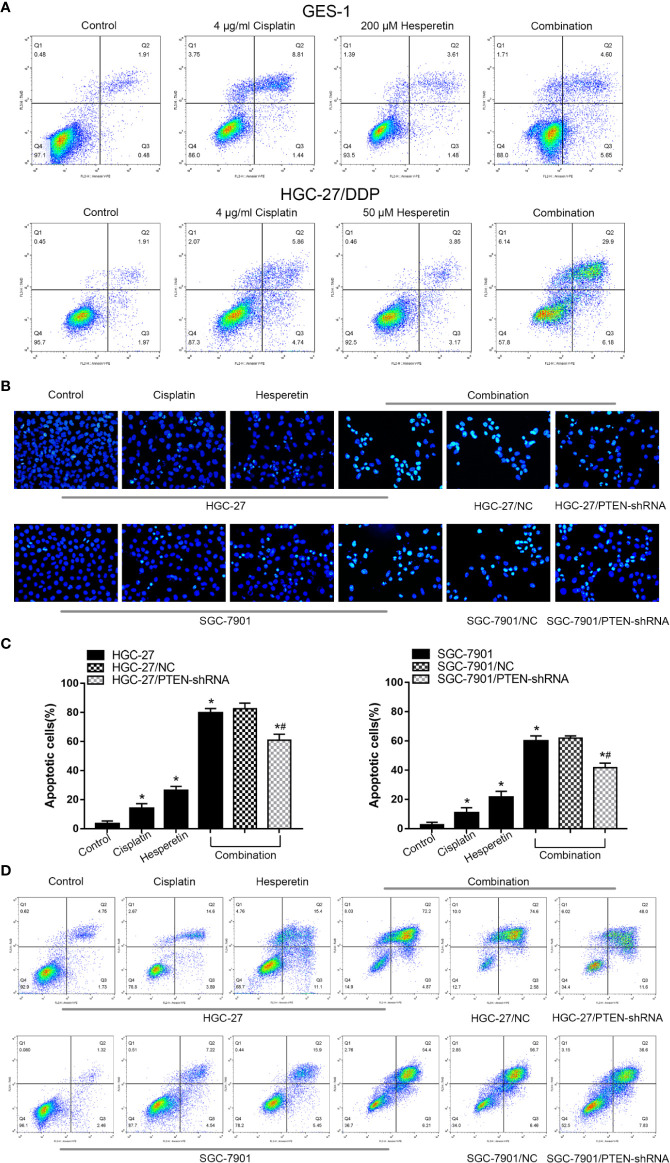
Hesperetin promotes the cisplatin-induced apoptosis of GC cells. **(A)** Quantitative flow cytometry measurements of apoptosis in GES-1 (control, 4 μg/ml cisplatin, 200 µM hesperetin, or 4 μg/ml cisplatin + 200 µM hesperetin) and HGC-27/DDP (control, 4 μg/ml cisplatin, 50 µM hesperetin, or 4 μg/ml cisplatin + 50 µM hesperetin) **(B)** Original magnification: 200×. HGC-27 (SGC-7901) cells were treated with control, 4 μg/ml cisplatin, 200 µM hesperetin, or 4 μg/ml cisplatin + 200 µM hesperetin; HGC-27(SGC-7901)/NC and HGC-27(SGC-7901)/PTEN-shRNA were treated with 4 μg/ml cisplatin + 200 µM hesperetin. **(C)** Quantitative analysis of the apoptosis rate in each group. *P < 0.05 *vs.* the control; *^#^*P* < 0.05 *vs.* NC cells. **(D)** Quantitative flow cytometry measurements of apoptosis in GC cells. All the above data are the mean ± SD from an average of three experiments.

To assess the morphology of apoptotic cells, Hoechst 33258 staining was used. Under a fluorescence microscope, normal-blue fluorescence could be observed in the control cells, while pyknosis and karyorrhexis with bright-blue fluorescence was observed in apoptotic cells. As shown in **Figure 3A**, 200 μM hesperetin has no obvious toxicity to GES-1, and the combination with DDP could not enhance the toxicity of DDP to normal cells. For DDP-resistant GC cells HGC-27/DDP, the combination of 50 μM hesperetin and DDP could significantly enhance the sensitivity of HGC-27/DDP to DDP. The results also showed that compared with the untreated control group, significantly more apoptotic cells were induced by DDP and/or hesperetin in HGC-27 and SGC-7901 cells. In addition, the rate of apoptosis in GC cells was also apparently higher in the combined treatment group than in the drug alone treatment groups (*P < 0.05). However, in the cells in which *PTEN* expression was downregulated *via* transfection with PTEN-shRNA, the combined treatment group showed a distinctly lower apoptotic rate compared with that of the NC group **(****Figures 3B, C****)**. These results suggested that hesperetin sensitized GC cells to apoptosis induced by DDP, which might be related to PTEN expression.

To further confirm the GC cell apoptosis induced by DDP and hesperetin, Annexin V-PE/7-AAD staining was used. As shown in the **Figure 3D**, HGC-27 and SGC-7901 cells treated with DDP (4 μg/ml) plus hesperetin (200 μM) comprised increased proportions of early and late apoptotic cells compared with those in the untreated control group, the DDP treatment group, and hesperetin treatment group, while downregulation of PTEN attenuated the drug combination effect.

### Hesperetin Enhances the Sensitivity of GC Cells to DDP by Upregulating PTEN Expression to Inhibit PI3K/AKT Signaling and Activate the Mitochondrial Pathway

The detailed mechanism of the hesperetin-mediated increase in the sensitivity of GC cells to DDP was investigated using western blotting to detect the abundances of related proteins. The PI3K/AKT signaling pathway is an important signaling pathway that controls the proliferation of cancer cells.

**Figure 4:**
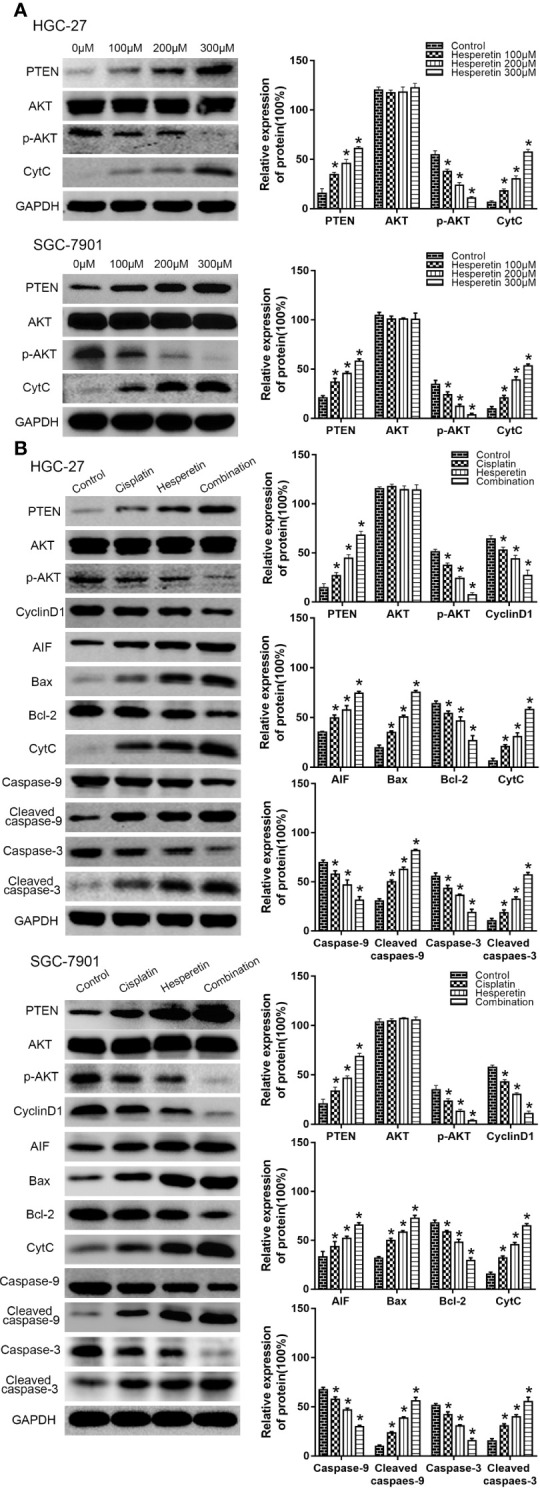
Hesperetin and cisplatin promote the apoptosis of GC cells *via* PTEN-PI3K/AKT and mitochondrial pathways. (**A)** HGC-27 (SGC-7901) cells were incubated with hesperetin (0, 100, 200, and 300 μM) for 24 h, and western blotting was performed to detect the levels of PTEN, AKT, p-AKT, and CytC. *P < 0.05 *vs.* the control group. **(B)** HGC-27 (SGC-7901) cells were incubated with the control, 4 μg/ml cisplatin, 200 μM hesperetin, or 4 μg/ml cisplatin + 200 μM hesperetin, and the levels of related proteins were measured using western blotting. *P < 0.05 *vs.* the control group. All the above data are the mean ± SD from an average of three experiments.

First, we investigated the underlying mechanism of hesperetin-induced cytotoxic effects on GC cells. HGC-27 and SGC-7901 cells were treated with hesperetin (0, 100, 200, and 300 μM) for 24 h. With increasing hesperetin concentration, the levels of PTEN and CytC increased significantly, while the level of p-AKT decreased distinctly, which indicated that hesperetin could activate the mitochondrial pathway and negatively regulate the PI3K/AKT pathway by upregulating PTEN expression (**Figure 4A****)**.

Then, we further studied the potential mechanism of the combined effect of DDP and hesperetin on GC cells. PTEN level increased significantly in the combination group. There was also an obvious increase in AIF and CytC levels, which were released from mitochondria into the cytoplasm when the mitochondrial membrane was damaged. In addition, although there was no significant difference in the level of total AKT, the p-AKT level was clearly reduced, especially in the combination group. Meanwhile, the level of Cyclin D1, an important downstream cell cycle protein of PI3K/AKT signaling, was also suppressed. We also observed decreases in the level of anti-apoptotic protein BCL2 in GC cells, whereas the abundance of the pro-apoptotic protein BAX increased obviously. In addition, in the combination group, a distinct increase in cleaved caspase-3 and cleaved caspase-9 levels, and a marked decrease in total caspase-3 and caspase-9 levels were observed compared with those in the DDP or hesperetin alone treatments **(****Figure 4B****)**.

Collectively, these results suggested that hesperetin enhances the apoptosis of GC cells induced by DDP by activating the mitochondrial pathway and inhibiting the PI3K/AKT pathway *via* increased PTEN expression.

### Downregulation of PTEN Expression Attenuates the Apoptosis Induced by DDP, Hesperetin Alone, and DDP Combined With Hesperetin

**Figure 5:**
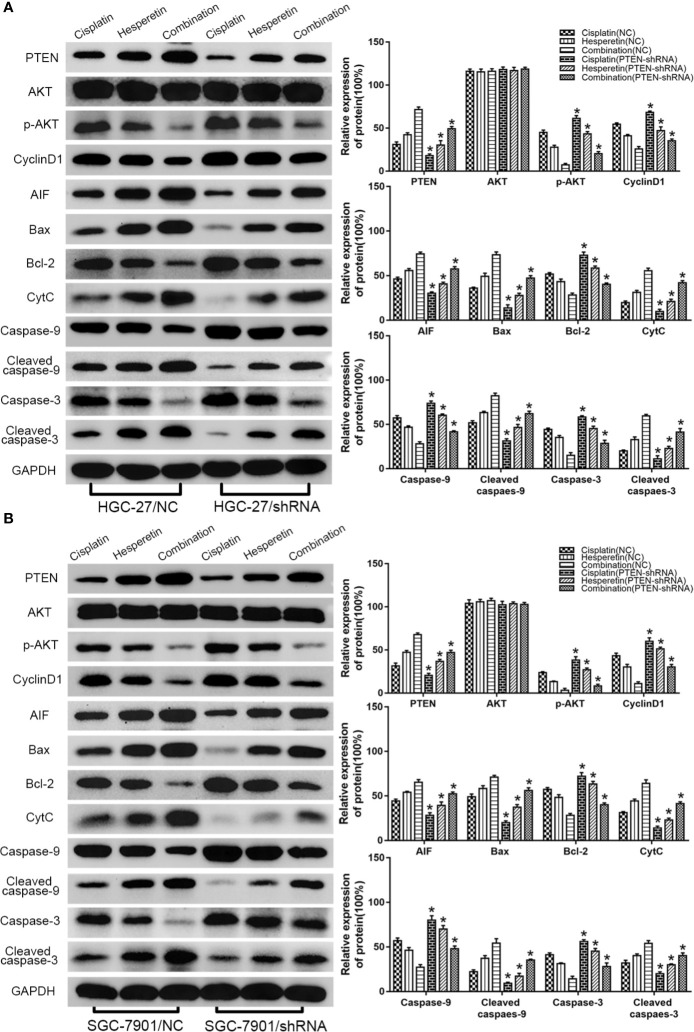
Downregulation of PTEN attenuates the apoptosis of GC cells induced by cisplatin and cisplatin combined with hesperetin. **(A)** HGC-27/NC and HGC-27/PTEN-shRNA cells were treated with 4 μg/ml cisplatin, 200 μM hesperetin, and 4 μg/ml cisplatin + 200 μM hesperetin, and western blotting was performed to detect the levels of related proteins. *P < 0.05 *vs.* the corresponding NC cells. **(B)** SGC-7901/NC and SGC-7901/PTEN-shRNA cells were also treated with cisplatin, hesperetin, and cisplatin + hesperetin, as described above; western blotting was performed to detect the levels of related proteins. *P < 0.05 *vs.* the corresponding NC cells. All the above data are the mean ± SD from an average of three experiments.

We further evaluated the effect of PTEN on the apoptosis of GC cells induced by DDP, hesperetin, and DDP combined with hesperetin. HGC-27(SGC-7901)/PTEN-shRNA and HGC-27(SGC-7901)/NC cells were treated with DDP alone (4 μg/ml), hesperetin alone (200 μM), and 4 μg/ml DDP + 200 μM hesperetin, respectively. As shown in **Figures 5A, B**, DDP + hesperetin caused an increase in the levels of PTEN, BAX, AIF, CytC, cleaved caspase-3, and cleaved caspase-9, and a marked decrease in the levels of p-AKT, Cyclin D1, and BCL2 compared with that of DDP or hesperetin alone in NC or PTEN-shRNA cells. However, compared with NC cells treated with 4 μg/ml DDP + 200 μM hesperetin or DDP/hesperetin alone, downregulation of PTEN resulted in a significant increase in p-AKT, Cyclin D1, and BCL2, and an apparent reduction in the levels of in BAX, AIF, CytC, cleaved caspase-3, and cleaved caspase-9 **(****Figures 5A, B****)**. These results suggested that hesperetin targeted *PTEN* to increase DDP-induced apoptosis of HGC-27(SGC-7901)/NC cells, thereby negatively regulating the PI3K/AKT signaling pathway and activating the mitochondrial apoptosis pathway. Furthermore, PTEN downregulation attenuated the apoptosis induced by DDP or hesperetin alone and DDP + hesperetin.

### *In Vivo* Anti-Tumor Effects of DDP and hesperetin on GC Cells

**Figure 6:**
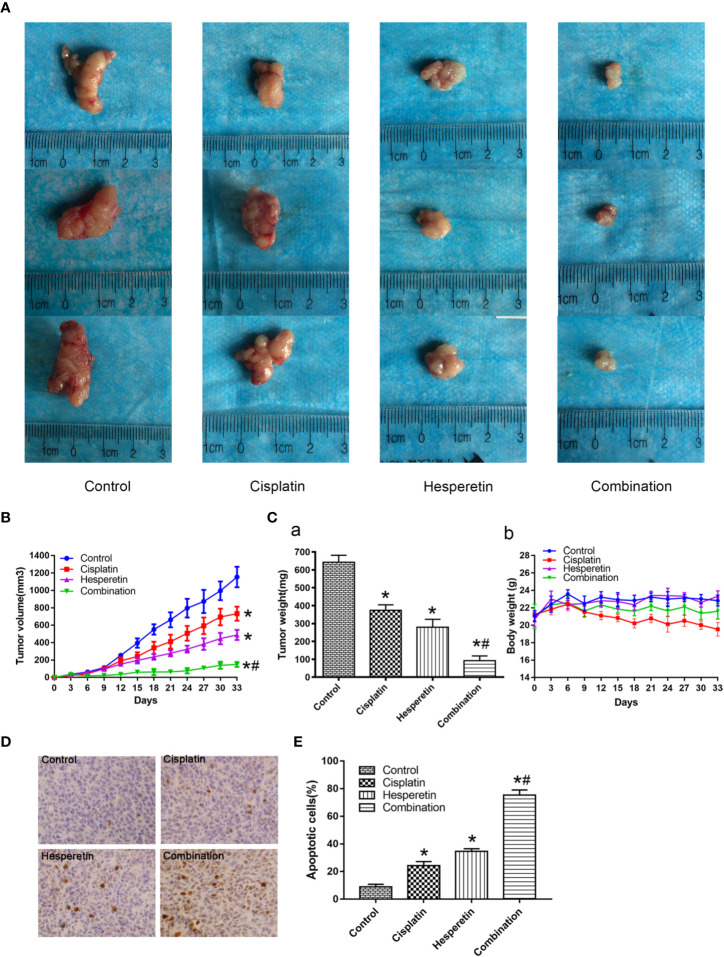
Anti-tumor effects of cisplatin and hesperetin *in vivo*. **(A)** Morphology of the subcutaneous implanted tumor. **(B)** Mean tumor volume at each time point. **(C) a**. The tumor weight obtained at the end of the experiment. **b**. The recorded body weights of the mice. **(D)** A TUNEL assay was performed to detect the apoptotic cells in the tumor tissue. **(E)** Quantitative analysis of the apoptosis rate in **(D)**. *P < 0.05 *vs.* the control, *^#^P < 0.05 *vs.* cisplatin alone. All the above data are the mean ± SD from an average of three experiments.

Based on the above *in vitro* experiments, hesperetin’s role in enhancing the anti-tumor effect of DDP was further investigated using a xenograft tumor model. The results showed that hesperetin and DDP, either alone or combined, induced strong *in vivo* inhibitory effects, resulting in significantly reduced tumor volume and weight in the treatment group **(****Figures 6A–Ca****)**. Furthermore, the combination of hesperetin and DDP resulted in a larger decrease in tumor volume and weight compared with that induced by hesperetin or DDP alone **(****Figures 6A–Ca****)**. DDP alone could reduce the body weight, while the combination of hesperetin and DDP had little effect on weight changes. Compared with the control group, hesperetin alone had no effect on the weight of the mice **(Figure 6Cb)**.

Tumor tissues isolated from the four groups of xenograft mice were subjected to a TUNEL assay and HE staining (not shown). TUNEL staining showed obvious apoptosis in the tumor from the treatment group; however the degree of apoptosis was different in each group. Compared with hesperetin or DDP alone, the tumor showed significantly more apoptosis in the combined group (**Figures 6D, E****)**. Liver and kidney injury was also assessed by measuring serum BUN, AST, ALT, and Cr levels; however, no significant difference was detected among the groups **(****Table 2****, P>0.05)**.

**Table 2:** Effect of hesperetin combined with cisplatin or alone on hepatic and renal function.

Group	ALT (U/l)	AST (U/l)	Urea (μmol/l)	Cr (μmol/l)
Control	32.67 ± 3.01	145.00 ± 12.31	8.22 ± 0.26	13.33 ± 2.42
Cisplatin	36.17 ± 4.45	141.50 ± 8.78	8.67 ± 0.89	14.50 ± 1.87
Hesperetin	34.50 ± 4.85	147.17 ± 15.80	8.57 ± 1.05	13.83 ± 1.94
Combination	34.33 ± 2.34	143.83 ± 3.31	8.46 ± 0.43	14.17 ± 2.23

Data are presented as the mean ± standard deviation, with n = 6 mice/group. No differences in the ALT, AST, Urea and Cr levels among all groups (P>0.05).

## Discussion

GC is prevalent throughout the world, especially in certain Asian countries. In China, GC mortality ranked second among cancer-related deaths in 2015 (Chen et al., 2016). In the clinic, drug side effects, drug resistance, and cancer recurrence often occur, resulting in poor prognosis for patients with GC (Shitara et al., 2018). The broad-spectrum anti-cancer agent, DDP, has been used widely for anti-tumor therapy, including the treatment of patients with GC. However, drug resistance associated with DDP treatment result in an obvious reduction in its efficacy (Ma et al., 2016; Hansen et al., 2017). Therefore, it is necessary and urgent to develop an efficient and safe drug, and new drug combinations. Our research group has demonstrated that increasing concentrations of hesperetin significantly inhibited the proliferation of cancer cells *in vitro* and *in vivo* (Zhang et al., 2015; Wu et al., 2016). More importantly, hesperetin is not only a potential anti-cancer drug, but also serves as a chemical sensitizer (Coutinho et al., 2017; Lee et al., 2019; Wang et al., 2019). Wang et al. reported that hesperetin was likely to act as an accelerator for the anti-cancer effect of DDP in lung adenocarcinoma (Wang et al., 2019). Although there is growing evidence that hesperetin induces apoptosis in different types of cancer cells (Ersoz et al., 2019; Li et al., 2019), whether hesperetin can enhance the chemical sensitivity of GC to DDP, and its specific mechanism, remains unclear.

PI3K/AKT signaling is an important regulatory pathway for the occurrence and development of cancer, regulating many cell activities, such as cell cycle progression, proliferation, and apoptosis (Warfel and Kraft, 2015; Spangle et al., 2017). Active PI3K mediates the phosphorylation of AKT at Thr 308 and Ser 473, resulting in partial or complete activation of AKT, respectively (Abraham, 2015). Activated AKT controls a variety of biological reactions, such as inhibiting apoptosis by directly phosphorylating apoptotic signal proteins or regulating the activity of transcription factors (Shaw and Cantley, 2006). Currently, it is generally believed that PTEN, as the main inhibitor of the PI3K/AKT signaling pathway, can negatively regulate the signaling pathway by dephosphorylating PIP3 to generate PIP2, which results in the downregulation of p-AKT, thus inhibiting the proliferation of cancer cells (Tay et al., 2011; Russell et al., 2018). Interestingly, Zhang et al. reported that Wortmannin sensitized DDP-resistant human lung cancer cells by downregulating the PI3K/AKT signaling pathway (Zhang et al., 2016), which prompted us to question whether hesperetin could also enhance the sensitivity of GC cells to DDP by inhibiting this pathway. Furthermore, a recent study showed that hesperetin inhibited the PI3K/AKT signaling pathway by upregulating PTEN expression, thus inhibiting cell proliferation (Li et al., 2017). Meanwhile, mutations in PTEN were observed in the occurrence and development of GC (Guo et al., 2013; Shen et al., 2018; Cai et al., 2019). Thus, we speculated that PTEN might have an important function in hesperetin-mediated sensitization of GC cells to DDP. BAX and BCL2 are downstream effectors of PI3K/AKT signaling that have important functions in the mitochondrial apoptosis pathway (Cosentino and Garcia-Saez, 2017). After activation, BAX migrates on the outer membrane of mitochondria in response to apoptosis stimulation, which is the key step to initiate apoptosis (Pena-Blanco and Garcia-Saez, 2018). Moreover, BAX can also promote the release of CytC from the mitochondria into the cytoplasm to activate multiple caspases, thus inducing cancer cell apoptosis (Cosentino and Garcia-Saez, 2017).

In the present study, the growth of GC cells was inhibited in a time-and dose-dependent manner by hesperetin combined with DDP **(****Figure 1****)**. Meanwhile, DDP-induced inhibition of GC cell invasion and migration, and the promotion of apoptosis, were enhanced by hesperetin; however, these effects were reversed by downregulation of PTEN **(****Figures 2** and **3****)**. Furthermore, in xenograft tumor models, DDP combined with hesperetin resulted in a significant decrease in tumor growth and a marked increase in the apoptosis of the tumor cells, with no obvious increase in adverse reactions **(****Figure 6****)**.

To explore whether hesperetin enhanced the sensitivity of GC cells to DDP by inhibiting the PTEN-PI3K/AKT pathway and activating the mitochondrial pathway, western blotting was used to detect the levels of corresponding proteins **(****Figure 4****)**. Compared with hesperetin or DDP alone, the level of PTEN in the DDP + hesperetin group was significantly higher, and the levels of p-AKT and CyclinD1 were obviously lower. In addition, it has been reported that the mitochondrial permeability transition pore (MPTP) plays an important role in mitochondrial-induced apoptosis, and BCL2 family proteins are one of its important components (Rasola and Bernardi, 2007). A decreasing BCL2/BAX ratio changes the permeability and structure of MPTP, which leads to the rupture of mitochondria and the release of CytC (Chen et al., 2015; McArthur et al., 2018). In addition, mitochondria release AIF into the cytoplasm, which then translocates to the nucleus, where it mediates chromatin condensation, resulting in cell death (Lorenzo and Susin, 2007; Bano and Prehn, 2018). Therefore, we further detected the levels of related proteins involved in the mitochondrial pathway, which showed that DDP combined with hesperetin resulted in significantly enhanced levels of BAX, AIF, CytC, cleaved caspase-3, and cleaved caspase-9, and significantly reduced the levels of caspase-3, BCL2, and caspase-9.

To further confirm the role of PTEN in the sensitization of GC cells to DDP induced by hesperetin, a PTEN-shRNA was stably transfected into GC cells to downregulate PTEN, with the NC plasmid as the control (Wu et al., 2008). Then, transfected GC cells were incubated with DDP, hesperetin or DDP + hesperetin. The results showed that hesperetin enhanced the growth inhibition and apoptosis of GC cells induced by DDP, while downregulation of PTEN reversed this effect **(****Figures 1F** and **3A****)**. Furthermore, compared with NC cells, there was a significant increase in p-AKT, CyclinD1, and BCL2 levels, and an apparent reduction in cleaved caspase-3, cleaved caspase-9, and apoptotic protein BAX levels in the PTEN shRNA transfected cells **(****Figure 5****)**.

In conclusion, the results provide strong molecular evidence to support the view that, both *in vitro* and *in vivo*, hesperetin enhances the anti-tumor effects induced by DDP on GC cells by activating the mitochondrial pathway and negatively regulating the PI3K/AKT signaling pathway by upregulating PTEN expression. Hesperetin combined with DDP shows great potential as a treatment for GC and merits further clinical investigation.

## Data Availability Statement

The raw data supporting the conclusions of this article will be made available by the authors, without undue reservation, to any qualified researcher.

## Ethics Statement

The animal study was reviewed and approved by Ethics Committee of Renmin Hospital of Wuhan University.

## Author Contributions

Conceptualization: PH and JM. Data curation: PH. Formal analysis: JM. Investigation: PH, YL, and HD. Methodology: JM. Project administration: PH and YL. Supervision: JM. Validation: YL and HD. Visualization: JM. Writing—original draft: PH. Writing—review and editing: WD.

## Funding

A research grant from the National Natural Science Foundation of China (No. 81572426) and the Fundamental Research Funds for the Central Universities (No. 2042020kf0107) supported this research.

## Conflict of Interest

The authors declare that the research was conducted in the absence of any commercial or financial relationships that could be construed as a potential conflict of interest.
